# The solid component within part-solid nodules: 3-dimensional quantification, correlation with the malignant grade of nonmucinous pulmonary adenocarcinomas, and comparisons with 2-dimentional measures and semantic features in low-dose computed tomography

**DOI:** 10.1186/s40644-023-00577-4

**Published:** 2023-06-22

**Authors:** Jieke Liu, Chaolian Xie, Yong Li, Hao Xu, Changjiu He, Haomiao Qing, Peng Zhou

**Affiliations:** Department of Radiology, Sichuan Clinical Research Center for Cancer, Sichuan Cancer Hospital & Institute, Sichuan Cancer Center, Affiliated Cancer Hospital of University of Electronic Science and Technology of China, Chengdu, China

**Keywords:** Part-solid nodule, Pulmonary adenocarcinoma, Solid component, Malignant grade, Low-dose computed tomography

## Abstract

**Background:**

There is no consensus on 3-dimensional (3D) quantification method for solid component within part-solid nodules (PSNs). This study aimed to find the optimal attenuation threshold for the 3D solid component proportion in low-dose computed tomography (LDCT), namely the consolidation/tumor ratio of volume (CTRV), basing on its correlation with the malignant grade of nonmucinous pulmonary adenocarcinomas (PAs) according to the 5th edition of World Health Organization classification. Then we tested the ability of CTRV to predict high-risk nonmucinous PAs in PSNs, and compare its performance with 2-dimensional (2D) measures and semantic features.

**Methods:**

A total of 313 consecutive patients with 326 PSNs, who underwent LDCT within one month before surgery and were pathologically diagnosed with nonmucinous PAs, were retrospectively enrolled and were divided into training and testing cohorts according to scanners. The CTRV were automatically generated by setting a series of attenuation thresholds from − 400 to 50 HU with an interval of 50 HU. The Spearman’s correlation was used to evaluate the correlation between the malignant grade of nonmucinous PAs and semantic, 2D, and 3D features in the training cohort. The semantic, 2D, and 3D models to predict high-risk nonmucinous PAs were constructed using multivariable logistic regression and validated in the testing cohort. The diagnostic performance of these models was evaluated by the area under curve (AUC) of receiver operating characteristic curve.

**Results:**

The CTRV at attenuation threshold of -250 HU (CTRV_− 250HU_) showed the highest correlation coefficient among all attenuation thresholds (*r* = 0.655, *P* < 0.001), which was significantly higher than semantic, 2D, and other 3D features (all *P* < 0.001). The AUCs of CTRV_− 250HU_ to predict high-risk nonmucinous PAs were 0.890 (0.843–0.927) in the training cohort and 0.832 (0.737–0.904) in the testing cohort, which outperformed 2D and semantic models (all *P* < 0.05).

**Conclusions:**

The optimal attenuation threshold was − 250 HU for solid component volumetry in LDCT, and the derived CTRV_− 250HU_ might be valuable for the risk stratification and management of PSNs in lung cancer screening.

## Background

With the widespread use of low-dose computed tomography (LDCT) in lung cancer screening, the detection of subsolid nodules is escalating. Most pathologically confirmed subsolid nodules are nonmucinous pulmonary adenocarcinomas (PAs) [1, 2]. which are divided into part-solid nodules (PSNs) and pure ground-glass nodules (PGGNs) on computed tomography (CT) [3]. The ground-glass opacity and solid component within PSNs tend to respectively correspond to lepidic and other invasive patterns in pathology, but this correlation is not absolute [4], resulting in no consensus on how to define and quantify the solid component of these lesions.

Previous clinical trials proposed a manual and 2-dimentional (2D) measure, consolidation-to-tumor ratio (CTR), to quantify the solid component, and it was employed to guide the mode of surgical resection [5–7]. It was also demonstrated that a higher CTR was associated with a poor prognosis in early-stage PAs [8, 9]. However, accurate and reproducible recognition and measurement of the solid component is challenging due to inter- and intra-observer variability using the subjective criterion [10, 11]. To eliminate the variability, many researchers have attempted to use objective, computer-aided, and 3-dimensional (3D) method instead of subjective, manual, and 2D method to measure the solid component. But the attenuation threshold for solid component was inconsistent, ranging from − 350 HU to -50 HU [12–15].

In the updated World Health Organization (WHO) classification of thoracic tumors, nonmucinous PA is classified into minimally invasive adenocarcinoma (MIA) and invasive PA (IPA), and IPA is further classified into well, moderately, and poorly differentiated IPA basing on the predominant and high-grade patterns [16]. This novel grading system for IPA offers a superior prognostic stratification compared with the previous classification system [17–20]. Specifically, MIA and well differentiated IPA (Grade 1), with lepidic predominant pattern and no or less than 20% of high-grade pattern, have a 5-year recurrence-free survival (RFS) rate of nearly 100%, while the moderately (Grade 2) to poorly (Grade 3) differentiated IPA have a 5-year RFS rate of 82.6–22.0%. Besides, high-grade IPAs could benefit from adjuvant treatment after surgical resection [21], such as chemotherapy, targeted therapy, and immunotherapy [22–25]. Thus preoperatively stratifying the malignant grade of nonmucinous PAs might guide the selection of personalized therapy. Until now, there are rare studies about the association between the solid component size within PSNs and the novel grading system. Besides, all previous studies used standard-dose CT rather than LDCT data [12–15], and thus the optimal attenuation threshold that could be used in lung cancer screening remained unaddressed.

Here we hypothesize that the 3D solid component proportion, namely the consolidation/tumor ratio of volume (CTRV), is associated with the malignant grade of nonmucinous PAs and can help stratify the risk of PSNs in lung cancer screening. Therefore, this study firstly aimed to find the optimal attenuation threshold for CTRV in LDCT basing on the correlation between the CTRV and the malignant grade of nonmucinous PAs. Secondly, we tested the ability of CTRV to distinguish between low-risk (MIA/Grade 1 IPA) and high-risk (Grade 2/3 IPA) nonmucinous PAs in PSNs, and compare its performance with 2D measures and semantic features.

## Methods

### Patients

This retrospective study was approved by the Institutional Review Board of Sichuan Cancer Hospital, and the need to obtain informed consent was waived. A total of 313 consecutive patients with **326** PSNs between November 2018 and May 2022 were enrolled in our institution. The inclusion criteria were: (a) patients with PSNs detected by LDCT in lung cancer screening; (b) first treatment with surgical resection; (c) pathologically diagnosed with nonmucinous PAs. The exclusion criteria were: (a) PGGNs or solid nodules; (b) patients who underwent LDCT scan over one month before surgery; (c) pathologically diagnosed with precursor glandular lesions, or variants of adenocarcinomas; (d) inadequate image quality due to respiratory and movement artifacts.

The whole sample of PSNs was divided into training and testing cohorts according to scanners. The training cohort (n = 239; MIA = 15; Grade 1 = 62; Grade 2 = 151; Grade 3 = 11) was used to determine the optimal attenuation threshold and construct the predicting models, whose generalization abilities were validated by the testing cohort (n = 87; MIA = 9; Grade 1 = 19; Grade 2 = 52; Grade 3 = 7).

### Acquisition parameters

The training cohort were obtained with a 64-detector dual-source CT scanner (Somatom Definition Flash, Siemens Healthcare) using the following acquisition parameters: tube voltage, 100 kV; tube current, 10 to 30 mAs; pitch, 1; collimation, 64 × 0.6 mm; rotation time, 0.33s; field of view, 350 mm × 350 mm. Then the images were reconstructed using the hybrid iterative reconstruction method (SAFIRE, Strength level 5) with soft reconstruction kernel (I30f), slice thickness of 0.5 mm, no gap, and matrix of 512 × 512. The estimated effective dose was 0.55 ± 0.11 mSv, and the mean interval between LDCT and surgery was 6.6 ± 5.2 days in the training cohort.

The testing cohort were obtained with a 128-detector CT scanner (Brilliance iCT, Philips Healthcare) using the following acquisition parameters: tube voltage, 100 kV; tube current, 20 to 30 mAs; pitch, 0.915; collimation, 128 × 0.625 mm; rotation time, 0.4s; field of view, 350 mm × 350 mm. Then the images were reconstructed using the hybrid iterative reconstruction method (iDose4, level 6) with soft reconstruction kernel (B), slice thickness of 0.625 mm, no gap, and matrix of 512 × 512. The estimated effective dose was 0.66 ± 0.12 mSv, and the mean interval between LDCT and surgery was 6.9 ± 5.4 days in the testing cohort.

### Histopathologic evaluation

The pathological diagnosis of nonmucinous PA was obtained according to the 5th edition of WHO classification of thoracic tumors [16]. The nonmucinous MIA was defined as a tumor of ≤ 3 cm with lepidic predominant growth and ≤ 5 mm of stromal invasion, no lymphatic, vascular or pleural invasion, and no tumor necrosis. The nonmucinous IPA was classified into three grades: well differentiated (Grade 1), lepidic predominant tumor, with no or less than 20% of high-grade pattern; moderately differentiated (Grade 2), acinar or papillary predominant pattern with no or less than 20% of high-grade pattern; poorly differentiated (Grade 3), any tumor with 20% or more of high-grade pattern. The high-grade pattern included micropapillary, solid, cribriform, and complex glandular patterns. The percentage of each histological pattern was recorded in 5% increments. In the secondary aim of this study, we divided nonmucinous PAs into low-risk group (MIA and Grade 1 IPA) and high-risk group (Grade 2/3 IPA) according to the distinct prognosis [17–20].

### Semantic features and 2D measures

Two radiologists (JL and HQ, with 6 years and 11 years of experience) who were blinded to histopathological diagnosis evaluated the semantic features and 2D measures of PSNs on LDCT images.

The semantic features included shape, margin, lobulation, spiculation, pleural indentation, air bronchogram, vacuole sign, and vascular convergence sign. The cases of disagreement between the two radiologists were resolved by consulting a third radiologist with 26 years of experience (PZ).

The 2D measures included the maximal diameter of nodule and CTR. The measurement of diameter followed the Fleischner Society guideline [3]. The CTR was defined as the ratio of the maximum diameter of solid component to the maximal diameter of nodule, which was in accordance with the definition of previous clinical trial [7]. The intra-class correlation coefficient (ICC) was used to evaluate the consistency of the 2D measures between two observers. ICC > 0.8 indicated a high agreement.

### 3D measures

The uAI platform (United Imaging Healthcare), an artificial intelligence (AI) software based on the deep learning method [26, 27], was used to automatically detect and segment pulmonary nodules in 3D. This software could be integrated into radiological diagnosis workflow by connecting with the picture archiving and communication system (PACS). It had been proved with satisfactory segmentation results in our previous studies [28–30]. To avoid inter- and intra-observer variability, no manual adjustment was conducted. The 3D measures including volume and mean attenuation were then automatically extracted by the AI software.

**Fig. 1:**
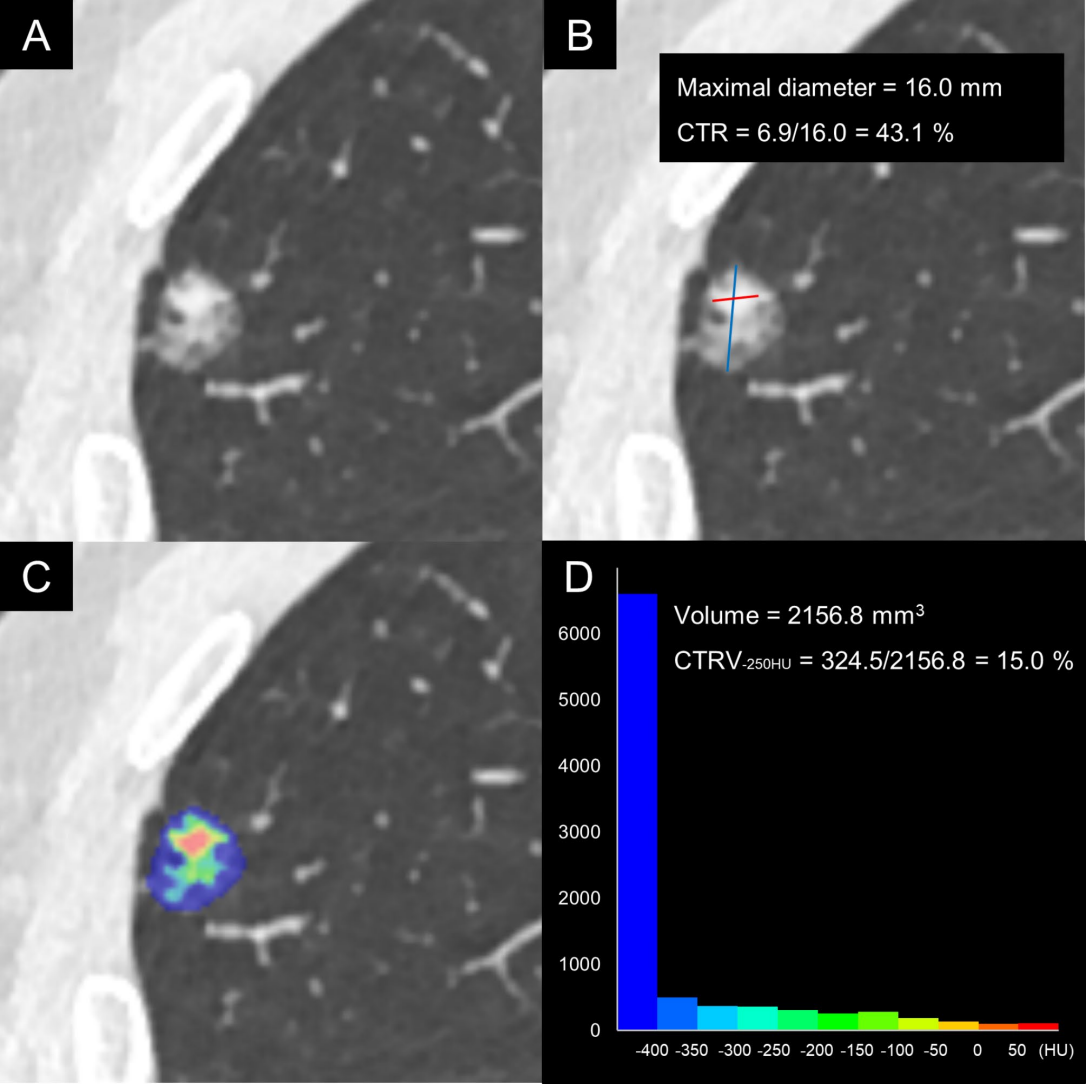
The quantification strategy of solid component within a representative part-solid nodule. A 56-year-old male patient with nonmucinous invasive pulmonary adenocarcinoma (Grade 2) in right upper lobe. **A**, Lung window setting reveals a part-solid nodule. **B**, Manual and 2-dimensional measurement of consolidation/tumor ratio (CTR) basing on the maximal diameters of nodule and solid component. **C**, Automatic segmentation and greyscale discretization of nodule (attenuation threshold ranges from − 400 to 50 HU, step = 50 HU). **D**, 3-dimensional histogram and measurement of consolidation/tumor ratio of volume (CTRV) at attenuation threshold of -250 HU basing on the volumes of nodule and solid component

After segmentation, a series of attenuation thresholds were set to conduct greyscale discretization, ranging from − 400 to 50 HU with an interval of 50 HU, which had been validated in a previous study [15]. The voxel volume beyond each attenuation threshold was automatically generated and recorded. The CTRV was defined as the ratio of the volume of solid component to the volume of nodule. The 2D and 3D quantification strategy of solid component within a representative PSN was shown in Fig. 1.

### Statistical analysis

Statistical analyses were performed with SPSS software (version 25.0; https://www.ibm.com) and Medcalc (version 18.2.1; https://www.medcalc.org/). The group differences were compared through Kruskal-Wallis test or Mann-Whitney *U* test for continuous variables, and Fisher’s exact test for categorical variables. The Spearman’s correlation was used to evaluate the association between the malignant grade of nonmucinous PAs and semantic features, 2D measures, and 3D measures. The attenuation threshold that showed the highest correlation coefficient between the malignant grade of nonmucinous PAs and the CTRV was selected as the optimal value for solid component volumetry. Statistical tests of differences between correlation coefficients were performed using the Fisher’s *Z*-transform method. A two-side *P* < 0.05 was considered statistically significant.

### Model construction and validation

We intended to construct three models, including semantic, 2D, and 3D models, to differentiate low-risk (MIA/Grade 1 IPA) from high-risk (Grade 2/3 IPA) nonmucinous PAs in PSNs. The potential predicting factors were firstly identified by group comparisons with statistical significance in the training cohort. Then, multivariable logistic regressions with backward stepwise selection were performed to constructed models by inputting these potential predicting factors in the training cohort. Finally, these models were validated in the testing cohort.

The area under curve (AUC) of receiver operating characteristic (ROC) curve was used to evaluate the performance of models. The optimal cut-off value was determined by Youden’s index and the corresponding sensitivity and specificity were calculated from the confusion matrix. The binomial exact method was used to estimate the 95% confidence intervals (CI). The comparisons of AUCs in the training and testing cohorts were performed using the Delong test [31]. The Hosmer-Lemeshow test was performed to evaluate the goodness-of-fit of models.

This study followed the transparent reporting of a multivariable prediction model for individual prognosis or diagnosis (TRIPOD) statement [32], and we concluded that the type of this study was type 2b.

## Results

### Demographic characteristics

The clinical and radiological characteristics of the training cohort in terms of the malignant grade of nonmucinous PAs were summarized in the Table 1. The age showed significant difference among the different malignant grade of nonmucinous PAs (*P* < 0.001). There was no significant difference in gender (*P* = 0.563). No significant correlation with the malignant grade of nonmucinous PAs was found in age (*r* = 0.009, *P* = 0.894) or gender (*r* = 0.088, *P* = 0.174).

**Table 1:** Characteristics of nonmucinous pulmonary adenocarcinomas that manifesting as part-solid nodules in the training cohort

Characteristic	MIA (n = 15)	IPA (n = 224)			*P*_1_^a^	*r*	*P*_2_^b^
		Grade 1 (n = 62)	Grade 2 (n = 151)	Grade 3 (n = 11)			
Gender					0.563	0.009	0.894
Male	4	24	48	5			
Female	11	38	103	6			
Age	47.5 ± 11.0	60.8 ± 9.3	58.5 ± 11.4	65.1 ± 10.8	< 0.001	0.088	0.174
Shape					0.999	0.007	0.917
Round/oval	0	2	4	0			
Irregular	15	60	147	11			
Margin					0.999	0.005	0.941
Smooth	0	1	2	0			
Coarse	15	61	149	11			
Lobulation					< 0.001	0.393	< 0.001
No	1	38	17	0			
Yes	14	24	134	11			
Spiculation					0.172	0.115	0.077
No	15	62	149	10			
Yes	0	0	2	1			
Pleural indentation					< 0.001	0.327	< 0.001
No	14	33	49	1			
Yes	1	29	102	10			
Air bronchogram					< 0.001	0.330	< 0.001
No	15	48	71	5			
Yes	0	14	80	6			
Vacuole					0.258	0.071	0.277
No	14	60	135	11			
Yes	1	2	16	0			
Vessel convergence					< 0.001	0.262	< 0.001
No	15	44	73	6			
Yes	0	18	78	5			
Maximal diameter (mm)	10.1 ± 2.5	17.6 ± 6.4	20.5 ± 7.0	24.0 ± 6.6	< 0.001	0.366	< 0.001
CTR (%)	38.6 ± 10.6	38.9 ± 13.1	53.4 ± 15.8	64.1 ± 14.1	< 0.001	0.456	< 0.001
Volume (mm^3^)	363.8 ± 266.0	2051.1 ± 2019.0	2770.3 ± 2305.2	3526.0 ± 2096.0	< 0.001	0.361	< 0.001
Mean attenuation (HU)	-598.3 ± 70.1	-578.5 ± 90.3	-426 ± 118.5	-331.4 ± 55	< 0.001	0.609	< 0.001
CTRV_− 400HU_ (%)	11.6 ± 7.6	17.5 ± 13.1	42.9 ± 19.6	57.5 ± 8.9	< 0.001	0.636	< 0.001
CTRV_− 350HU_ (%)	8.0 ± 5.6	13.5 ± 11.1	37.0 ± 18.9	51.7 ± 9.3	< 0.001	0.641	< 0.001
CTRV_− 300HU_ (%)	5.2 ± 4.1	10.1 ± 9.2	31.5 ± 18.0	46.1 ± 9.5	< 0.001	0.650	< 0.001
CTRV_− 250HU_ (%)	3.4 ± 2.9	7.5 ± 7.6	26.5 ± 16.7	40.6 ± 9.7	< 0.001	0.655	< 0.001
CTRV_− 200HU_ (%)	1.8 ± 1.8	5.4 ± 6.1	21.8 ± 15.4	35 ± 9.8	< 0.001	0.654	< 0.001
CTRV_− 150HU_ (%)	0.7 ± 1.2	3.7 ± 4.8	17.5 ± 13.9	29.5 ± 10.0	< 0.001	0.654	< 0.001
CTRV_− 100HU_ (%)	0.3 ± 0.7	2.5 ± 3.6	13.6 ± 12.2	24.0 ± 10.4	< 0.001	0.647	< 0.001
CTRV_− 50HU_ (%)	0.1 ± 0.2	1.4 ± 2.5	9.9 ± 10.4	18.7 ± 10.6	< 0.001	0.632	< 0.001
CTRV_0HU_ (%)	0.0 ± 0.0	0.7 ± 1.4	6.4 ± 8.1	13.0 ± 9.5	< 0.001	0.611	< 0.001
CTRV_50HU_ (%)	0.0 ± 0.0	0.2 ± 0.6	2.6 ± 4.1	5.6 ± 5.4	< 0.001	0.580	< 0.001

^a^ The group comparisons among different malignant grade of nonmucinous pulmonary adenocarcinomas. ^b^ The Spearman’s correlation analyses between characteristics and malignant grade of nonmucinous pulmonary adenocarcinomas. *MIA*, minimally invasive adenocarcinoma; *IPA*, invasive pulmonary adenocarcinoma; *CTR*, consolidation-to-tumor ratio; *HU*, Hounsfield unit; *CTRV*, consolidation/tumor ratio of volume

The clinical and radiological characteristics of the training and testing cohorts in terms of the low-risk and high-risk nonmucinous PAs were summarized in the Table 2. No significant difference was found in the gender or age in the training cohort or testing cohort (all *P* > 0.05).

**Table 2:** Characteristics of low-risk and high-risk nonmucinous pulmonary adenocarcinomas that manifesting as part-solid nodules in the training and testing cohorts

Characteristic	Training cohort		*P*	Testing cohort		*P*
	Low-risk (n = 77)	High-risk (n = 162)		Low-risk (n = 28)	High-risk (n = 59)	
Gender			0.661			0.487
Male	28	53		10	27	
Female	49	109		18	32	
Age	58.2 ± 10.9	59.0 ± 11.4	0.718	59.1 ± 10.6	58.2 ± 11.9	0.696
Shape			0.999			0.264
Round/oval	2	4		4	4	
Irregular	75	158		24	55	
Margin			0.999			0.591
Smooth	1	2		2	2	
Coarse	76	160		28	57	
Lobulation			< 0.001			0.074
No	39	17		12	13	
Yes	38	145		16	46	
Spiculation			0.553			0.548
No	77	159		28	56	
Yes	0	3		0	3	
Pleural indentation			< 0.001			0.036
No	47	50		16	19	
Yes	30	112		12	40	
Air bronchogram			< 0.001			0.051
No	63	76		23	35	
Yes	14	86		5	24	
Vacuole			0.130			0.999
No	74	146		23	48	
Yes	3	16		5	11	
Vessel convergence			< 0.001			0.036
No	59	79		25	40	
Yes	18	83		3	19	
Maximal diameter (mm)	16.1 ± 6.5	20.8 ± 7.0	< 0.001	15.3 ± 6.7	21.2 ± 6.3	< 0.001
CTR (%)	38.9 ± 12.6	54.1 ± 15.9	< 0.001	42.6 ± 14.3	57.7 ± 20.8	0.001
Volume (mm^3^)	1722.4 ± 1933.2	2821.6 ± 2293.5	< 0.001	1453.8 ± 1456.3	3217.4 ± 2585.6	< 0.001
Mean attenuation (HU)	-582.4 ± 86.7	-419.6 ± 117.6	< 0.001	-547.8 ± 91.8	-416.8 ± 124.8	< 0.001
CTRV_− 250HU_ (%)	6.7 ± 7.1	27.4 ± 16.7	< 0.001	8.7 ± 9.4	27.6 ± 17.3	< 0.001

*CTR*, consolidation-to-tumor ratio; *HU*, Hounsfield unit; *CTRV*, consolidation/tumor ratio of volume

### Semantic features

The lobulation, pleural indentation, air bronchogram, and vascular convergence sign showed significant difference among the different malignant grade of nonmucinous PAs (all *P* < 0.001), and significantly correlated with the malignant grade of nonmucinous PAs (*r* ranged from 0.262 to 0.393, all *P* < 0.001). No significant difference was found in the shape, margin, spiculation, or vacuole sign (all *P* > 0.05), and there is also no significant correlation with the malignant grade of nonmucinous PAs for these semantic features (*r* ranged from 0.005 to 0.115, all *P* > 0.05) (Table 1).

In the comparisons between the low-risk and high-risk nonmucinous PA groups, the pleural indentation and vascular convergence sign showed significant difference in both the training and testing (all *P* < 0.05) cohorts. The lobulation and air bronchogram showed significant difference in the training cohort (both *P* < 0.001), but no significant difference in the testing cohort (both *P* > 0.05). No significant difference was found in the shape, margin, spiculation, or vacuole sign in the training cohort or testing cohort (all *P* > 0.05) (Table 2).

### 2-dimensional measures

The ICC was 0.949 (0.938–0.959) for the maximal diameter and 0.940 (0.926–0.952) for the CTR in the combined training and testing cohorts, indicating a high agreement between the two radiologists. Then averages of the two radiologists were calculated for the following analysis.

The maximal diameter and CTR showed significant difference among the different malignant grade of nonmucinous PAs (both *P* < 0.001), and significantly correlated with the malignant grade of nonmucinous PAs (*r* = 0.366 and 0.456, both *P* < 0.001) (Table 1).

The high-risk nonmucinous PA group showed significantly higher maximal diameter and CTR than the low-risk group in both the training and testing (all *P* ≤ 0.001) (Table 2).

### 3-dimentinal measures

The volume, mean attenuation, and CTRVs at all attenuation thresholds showed significant difference among the different malignant grade of nonmucinous PAs (all *P* < 0.001), and significantly correlated with the malignant grade of nonmucinous PAs (*r* ranged from 0.580 to 0.655, all *P* < 0.001) (Table 1).

The CTRV at attenuation threshold of -250 HU (CTRV_− 250HU_) showed the highest correlation coefficient (*r* = 0.655) among all attenuation thresholds, which was also significantly higher than that of lobulation, pleural indentation, air bronchogram, vascular convergence sign, maximal diameter, CTR, volume, and mean attenuation (*Z* ranged from 3.290 to 6.676, all *P* < 0.001). Therefore, the attenuation of -250 HU was selected as the optimal threshold for solid component volumetry, and CTRV_− 250HU_ was used for the following analysis.

The high-risk nonmucinous PA group showed significantly higher volume, mean attenuation, and CTRV_− 250HU_ than the low-risk group in both the training and testing (both *P* < 0.001) (Table 2).

### Model construction

According to the group comparison results in the training cohort, the potential predicting factors for differentiating low-risk from high-risk nonmucinous PAs included four semantic features (lobulation, pleural indentation, air bronchogram, and vascular convergence sign), two 2D measures (maximal diameter and CTR), and three 3D measures (volume, mean attenuation, and CTRV_− 250HU_). Then, these factors were integrated to construct semantic, 2D, and 3D models using multivariable logistic regressions.

**Among four semantic features, vascular convergence sign was eliminated from the final model after backward stepwise selection (**Table 3**), and thus** the calculation formula for semantic model was: **ln [P/(1 − P)]** = -1.580 + 1.817 × lobulation _(yes)_ + 0.980 × pleural indentation _(yes)_ + 1.424 × air bronchogram _(yes)_, where P is the probability of high-risk nonmucinous PAs (cut-off > 0.627).

**Table 3:** Multivariable logistic regressions of selected features for differentiating low-risk from high-risk nonmucinous pulmonary adenocarcinomas in the training cohort

Models		Odds ratio	95% confidence interval	*P*
Semantic	Lobulation			< 0.001
	No ^a^	1		
	Yes	6.153	3.028–12.505	
	Pleural indentation			0.003
	No ^a^	1		
	Yes	2.655	1.400–5.075	
	Air bronchogram			< 0.001
	No ^a^	1		
	Yes	4.153	2.012–8.575	
2-dimensional	Maximal diameter	1.128	1.069–1.190	< 0.001
	CTR	1.082	1.055–1.110	< 0.001
CTRV_− 250HU_	CTRV_− 250HU_	1.176	1.123–1.231	< 0.001

^a^ Features were set as reference. *CTR*, consolidation-to-tumor ratio; *CTRV*, consolidation/tumor ratio of volume

For 2D model, both maximal diameter and CTR were retained in the final model after backward stepwise selection** (**Table 3), and thus the calculation formula for 2D model was: ln [P/(1 − P)] = -5.061 + 0.120 × maximal diameter + 0.079 × CTR, where P is the probability of high-risk nonmucinous PAs (cut-off > 0.676).

For 3D model, volume and mean attenuation were eliminated from the final model after backward stepwise selection, and thus only CTRV_− 250HU_ was retained in 3D model (Table 3). In order to conveniently use this index, there was no need to fit logistic function. The cut-off of CTRV_− 250HU_ for the high-risk nonmucinous PAs was 8.6%.

### Model performance

**Fig. 2:**
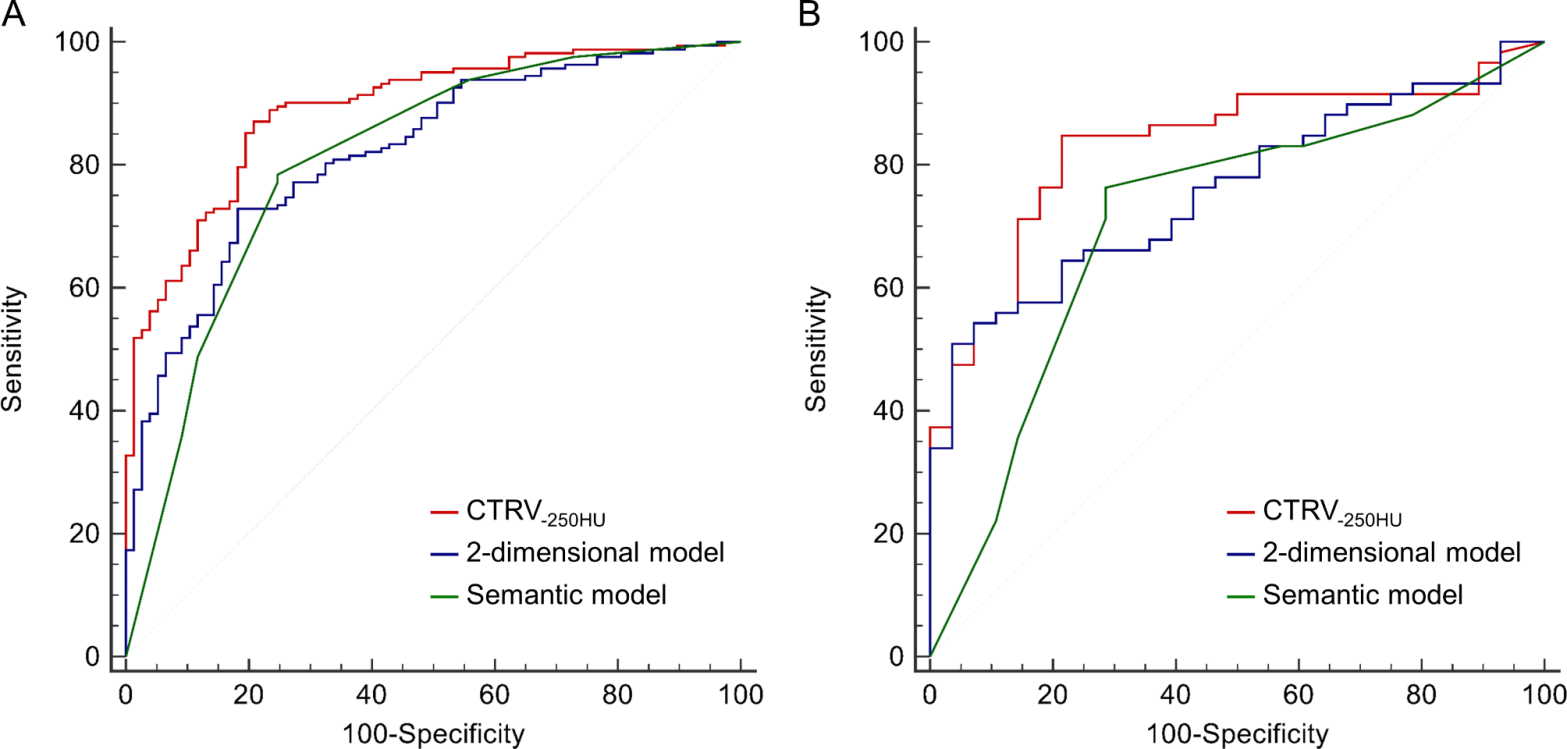
The receiver operating characteristic curves of semantic model, 2-dimensional model, and CTRV_− 250HU_ for differentiation between low-risk and high-risk nonmucinous pulmonary adenocarcinomas in part-solid nodules. **A**, Training cohort. **B**, Testing cohort

The ROC curves of semantic model, 2D model, and CTRV_− 250HU_ in the training and testing cohorts were shown in Fig. 2. The AUCs of semantic model, 2D model, and CTRV_− 250HU_ were 0.809 (0.753–0.856), 0.821 (0.767–0.868), and 0.890 (0.843–0.927) in the training cohort, and 0.710 (0.603–0.802), 0.760 (0.656–0.845), and 0.832 (0.737–0.904) in the testing cohort, respectively. The detailed sensitivity and specificity were present in Table 4.

**Table 4:** Diagnostic performance of models in the training and testing cohorts

Models	AUC (95% CI)	Sensitivity (95% CI)	Specificity (95% CI)	*Z*	*P* ^a^
Training cohort					
Semantic	0.809 (0.753–0.856)	0.784 (0.713–0.845)	0.753 (0.642–0.844)	2.755	0.006
2-dimensional	0.821 (0.767–0.868)	0.728 (0.653–0.795)	0.818 (0.714–0.897)	3.097	0.002
CTRV_− 250HU_	0.890 (0.843–0.927)	0.870 (0.809–0.918)	0.792 (0.685–0.876)	-	-
Testing cohort					
Semantic	0.710 (0.603–0.802)	0.763 (0.634–0.864)	0.714 (0.513–0.868)	1.990	0.047
2-dimensional	0.760 (0.656–0.845)	0.661 (0.526–0.779)	0.750 (0.551–0.893)	2.108	0.035
CTRV_− 250HU_	0.832 (0.737–0.904)	0.847 (0.730–0.928)	0.786 (0.590–0.917)	-	-

^a^ Comparison of AUC with 3-dimensional model. *AUC*, area under curve; *CI*, confidence interval

According to the DeLong test, the AUC of CTRV_− 250HU_ was significantly higher than that of semantic model and 2D model in the training cohort (*P* = 0.006 and 0.002) as well as in the testing cohort (*P* = 0.047 and 0.035). The Hosmer-Lemeshow test yielded non-significant results in both the training and testing cohorts of semantic model (*P* = 0.451 and 0.075), 2D model (*P* = 0.662 and 0.316), and CTRV_− 250HU_ (*P* = 0.410 and 0.184), which suggested no departure from the perfect fit.

## Discussion

In this study, we demonstrated that the 3D solid component proportion, namely the CTRV, was associated with the malignant grade of nonmucinous PAs in PSNs, and the optimal attenuation was − 250 HU for solid component volumetry in LDCT. Besides, the CTRV_− 250HU_ showed higher correlation with the malignant grade of nonmucinous PAs and better performance to predict high-risk nonmucinous PAs than 2D measures and semantic features in PSNs.

As the solid component was an indicator of invasiveness [33–35] and also correlated with the non-leipidic invasive component size at pathological examinations [36, 37], the guidelines of lung cancer screening recommended to manage PSNs on the basis of the solid component diameter on CT [3, 38, 39]. However, the inter-observer variability in measuring the diameter of pulmonary nodule could not be neglected, especially in the presence of a solid component [10, 11]. Besides, the current morphologic criteria for defining solid component had limited operability in lung cancer screening. According to the Fleischner recommendations, nodule components other than normal vascular or bronchial structures remained visible on thin sections with mediastinal (soft tissue) window settings and sharp filters were regarded as solid components [3]. But LDCT images were usually acquired by using smooth reconstruction kernels to improve the signal-to-noise ratio because the sharp kernels substantially increase image noise with negative effects on detail visibility [10]. Another method to determine the presence of solid component based on whether the maximum CT value of the subsolid nodule was higher than that of the vessel [15]. But it could not accurately define the margin and size of solid component. The automatic quantification method in our study, with adequate objectivity, might address the question that how to measure solid component volume in PSNs during lung cancer screening.

Many efforts had been triggered to measure solid component through 3D and automatic methods beyond 2D and manual methods. Our optimal attenuation threshold was − 250 HU, which was inconsistent with previous studies [12–15]. There were several possible reasons. The first was the using of different pathological standards to select the optimal threshold, such as the size of invasive component under light microscopy [13] and the ability to differentiate invasive from noninvasive PAs [14, 15]. The second possible reason might be the using of different threshold selection methods. The computer-based automatic thresholding and CT visual appearance-based thresholding methods might be short of biological basis and objectivity to some extent, respectively [12, 14]. Here we used the correlation between the 3D solid component proportion deriving from objective and automatic method and the malignant grade of nonmucinous PAs basing on the novel edition of WHO classification [16]. Thirdly, it was known that the reconstruction kernel and radiation dose significantly affected quantification of CT attenuation [40–43]. But there was the heterogeneity in these acquisition parameters among previous and our works. All the previous studies used standard-dose CT rather than LDCT. Besides, some images were reconstructed with sharp kernel [12, 13], while some were smooth kernel [14, 15].

Previous study found the volume had better ability to reflect the 3D nature of pulmonary nodules than diameter, as it allowed the calculation of volume doubling time to more reliably define the nodule growth and reduced subjective inconsistency among observers [44]. The Lung CT Screening Reporting & Data System (Lung-RADS, version 1.1) was also updated in 2019 to include volume in management of pulmonary nodules [38]. However, the solid component volume has not been included in any guideline or recommendation to manage PSNs, because there is still no consensus about the appropriate 3D quantification method for solid component. The CTRV_− 250HU_ in our study, a 3D measure of solid component proportion that showed higher correlation with the malignant grade of nonmucinous PAs and better performance to predict high-risk nonmucinous PAs than 2D measures, might be valuable for the risk stratification and management of PSNs in lung cancer screening.

Several morphologic features constituted the semantic model, including lobulation, pleural indentation, and air bronchogram, which were more common in the high-risk nonmucinous PA group than in the low-risk group in PSNs. The lobulation was generally attributed to different or uneven growth rates within nodules. The pleural indentation presented scar contraction caused by fibrotic hyperplasia, and active fibroblast proliferation was associated with the invasive growth of tumors [45, 46]. The air bronchogram was a pattern of air-filled bronchi against a background of airless lung. All these morphologic features were found to be related to invasiveness of PAs in PSNs [47–49]. However, in terms of predicting high-risk nonmucinous PAs in PSNs, the semantic model was inferior to the CTRV_− 250HU_ in our results.

In this study, we proposed a novel method that could be used to quantify solid component within PSNs in 3D during lung cancer screening. The advantage of this method was the feasibility and applicability in LDCT images with satisfactory objectivity. However, the optimal attenuation threshold was generated by a certain cohort, but not in an individualized level. Therefore, further researches may focus on how to automatically define the optimal attenuation threshold in a personalized manner. The developed deep learning-based AI tools, with excellent ability in detecting and segmenting pulmonary nodules at present [50], may have the potential to fill this gap. Recently, Ahn et al. found the deep learning algorithm could automatically measure the maximal diameter of solid component in 2D [37]. The AI tool, basing on big data and standardized 3D annotation of solid component, is needed to be developed and validated in the future.

Our study had limitations. First, due to the inherent nature of the retrospective study, the potential selection bias was unavoidable. The sample size was also relatively limited. Thus further prospective and large-scale trial is required to confirm the predicting performance of our models. Second, this was a single-center study. However, a testing dataset from another scanner was used to validate the proposed models, making them more convincing and reproducible. Third, the protocols of LDCT used in this study might not be applicable to other scanners and acquisition parameters. Therefore, further multi-center study is still needed to validate the generalizability of our models.

## Conclusions

This study indicated that the optimal attenuation threshold was − 250 HU for solid component volumetry in LDCT. The derived CTRV_− 250HU_ showed higher correlation with the malignant grade of nonmucinous PAs and better performance to predict high-risk nonmucinous PAs than 2D measures and semantic features in LDCT, which might be valuable for the risk stratification and management of PSNs in lung cancer screening.

## Data Availability

The datasets used and/or analyzed during the current study are available from the corresponding author on reasonable request.

## Abbreviations

2D: 2-dimentional
3D: 3-dimentional
AI: Artificial intelligence
AUC: Area under curve
CI: Confidence interval
CT: Computed tomography
CTR: Consolidation-to-tumor ratio
CTRV: Consolidation/tumor ratio of volume
ICC: Intra-class correlation coefficient
IPA: Invasive pulmonary adenocarcinoma
LDCT: Low-dose computed tomography
Lung-RADS: Lung CT Screening Reporting & Data System
MIA: Minimally invasive adenocarcinoma
PA: Pulmonary adenocarcinoma
PACS: Picture archiving and communication system
PGGN: Pure ground-glass nodule
PSN: Part-solid nodule
RFS: Recurrence-free survival
ROC: Receiver operating characteristic
TRIPOD: Transparent reporting of a multivariable prediction model for individual prognosis or diagnosis
WHO: World Health Organization
